# Remittances

**Published:** 1887-04

**Authors:** 


### REMITTANCES.

Many subscribers are exceedingly careless in making remittances. We not infrequently receive postoffice money orders that are payable only in New York City. Again, orders are sent to our New York office, although made payable in Buffalo. Many orders are sent without any letter of advice or means of identification, other than that contained in the letter of the postmaster at the sending office to the one at the paying office, and we are obliged to depend upon the courtesy of the latter to furnish the name of the subscriber to whom the money should be credited.

Occasionally we notice in the pages of some specially good-natured contemporary an allusion to this journal as being published in Buffalo. It should be thoroughly understood that the *publication* office is in New York. The editorial office is in Buffalo, and it is mailed from that city because it is more convenient to do so, but its business office is in New York, and there is where the majority of its publishers reside. The subscription books are kept in Buffalo, and the prospectus plainly states that all subscription bills should be paid there. Will subscribers please bear in mind, then, that *all money for subscriptions should be sent to Buffalo,* and all checks and money orders for that account should be made payable to the editor at that place. All letters upon other business matters, advertising, etc., should be directed to the New York office. "Paste this notice in your hat for future reference."

